# Proprioception and Mechanoreceptors in Osteoarthritis: A Systematic Literature Review

**DOI:** 10.3390/jcm12206623

**Published:** 2023-10-19

**Authors:** Francesca Salamanna, Silvio Caravelli, Laura Marchese, Melania Carniato, Emanuele Vocale, Giammarco Gardini, Giulia Puccetti, Massimiliano Mosca, Gianluca Giavaresi

**Affiliations:** 1 Surgical Sciences and Technologies, IRCCS Istituto Ortopedico Rizzoli, Via di Barbiano 1/10, 40136 Bologna, Italy; laura.marchese@ior.it (L.M.); melania.carniato@ior.it (M.C.); gianluca.giavaresi@ior.it (G.G.); 2 IRCCS Istituto Ortopedico Rizzoli, Via Pupilli 1, 40136 Bologna, Italy; silvio.caravelli@ior.it (S.C.); massimiliano.mosca@ior.it (M.M.); 3 2nd Orthopaedic and Traumatologic Clinic, IRCCS Istituto Ortopedico Rizzoli, Via Pupilli 1, 40136 Bologna, Italy; emanuele.vocale@ior.it (E.V.); giammarco.gardini@ior.it (G.G.); giulia.puccetti@studio.unibo.it (G.P.)

**Keywords:** osteoarthritis, proprioception, mechanoreceptors, clinical studies, systematic review

## Abstract

Purpose: Osteoarthritis (OA) is one of the most common chronic diseases in the world. It is frequently accompanied by high levels of persistent pain, as well as substantial impairments in function and functional capacity. This review aims to systematically analyze the changes in proprioception and related mechanoreceptors in OA patients. Methods: Studies from September 2013 to September 2023 were identified by conducting searches on the PubMed, Web of Science, and Scopus electronic databases following the PRISMA statement. One reviewer independently assessed and screened the literature, extracted the data, and graded the studies. The body of evidence underwent an evaluation and grading process using the ROBINS-I tool, which was specifically designed to assess the risk of bias in non-randomized studies of interventions. Results were summarized using descriptive methods. Results: A search through 37 studies yielded 14 clinical studies that were ultimately included. The primary focus of the studies was on the knee joint, particularly the posterior cruciate ligament (PCL). The studies found that PCL in OA patients had impaired proprioceptive accuracy, possibly due to changes in mechanoreceptors (Ruffini, Pacini, and Golgi Mazzoni corpuscles). This suggests that dysfunctional articular mechanoreceptors, especially in severe cases of OA, may contribute to reduced proprioception. Dynamic stabilometry also identified significant proprioceptive deficits in patients with knee articular cartilage lesions, underscoring the impact of such lesions on knee proprioception. Conclusions: Literature data have shown that proprioceptive accuracy may play an important role in OA, particularly in the knee PCL and cartilage. However, the role of proprioception and related mechanoreceptors needs to be further clarified. Future studies focusing on the relationship between proprioception, OA disease, and symptoms, considering age and gender differences, and exploring OA joints other than the knee should be conducted to improve clinical and surgical outcomes in cases where proprioception and mechanoreceptors are impaired in OA patients.

## 1. Introduction

Osteoarthritis (OA) is a persistent condition characterized by the degeneration of joints, and it affects a staggering number of individuals worldwide, with over 300 million people impacted [1]. It represents a complete organ failure, affecting not only the cartilage but also involving the entire joint [1,2,3]. It has become the third most rapidly rising condition associated with disability, following diabetes and dementia. Given the increasing life expectancy in numerous countries, it is anticipated that the global prevalence of OA will double by the year 2040 [2,3,4,5,6]. Recently, the impaired proprioceptive accuracy of joints has been proposed as a potential local factor in the onset and progression of OA [7,8]. Proprioceptive impairments could be a cause of joint pain or activity limitations in OA patients [7,8].

**Figure 1:**
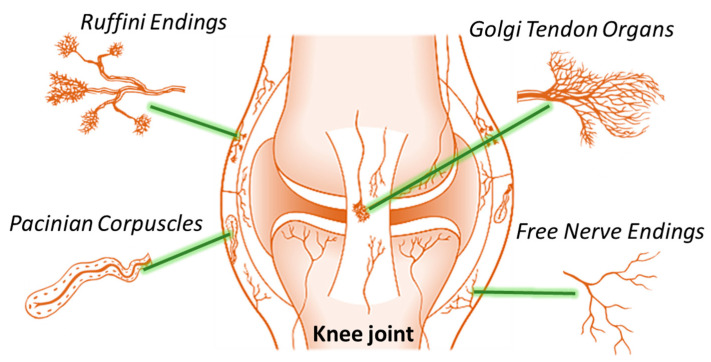
Schematic representation of the potential location of the four (free nerve endings, Ruffini Endings, Pacinian Corpuscles, Golgi Tendon Organs) joint mechanoreceptors. Adapted from Freeman et al. [12].

Mechanoreceptors play a central role in proprioception as they are sensory receptors responsible for detecting mechanical stimuli, such as pressure, stretch, and vibration, in various tissues, including muscles, tendons, ligaments, and joints [9,10]. There are several types of joint mechanoreceptors. Type I Receptors, or free nerve endings, are widespread and sensitive to mechanical changes in the joint, like movement and compression [9,10]. Type II Receptors, known as Ruffini Endings, are encapsulated nerve endings that respond to joint position and slow, sustained stretching. Type III Receptors, Pacinian Corpuscles, are rapidly adapting and detect high-frequency vibrations and joint pressure changes. Type IV Receptors, Golgi Tendon Organs, are located in ligaments and capsules, responding to alterations in joint tension and force. While there is ongoing debate about their precise types and locations, these mechanoreceptors play a crucial role in sensory input and coordination regulation within joints (Figure 1) [9,10,11].

In the context of OA, proprioceptive function and mechanoreceptors can be affected due to several factors: (1) OA leads to the loss and degradation of articular cartilage [13]. This loss of cartilage can disrupt the normal mechanics of the joint, leading to altered proprioceptive feedback. (2) OA is associated with joint inflammation, leading to increased joint fluid and swelling [14]. Swollen joints can alter the position and perception of the joint, affecting proprioception. (3) OA can also affect the ligaments and tendons surrounding the joint [15]. These structures are rich in mechanoreceptors and provide important proprioceptive input. Damage to ligaments and tendons in OA can impair proprioception. (4) As OA progresses, there might be muscle weakness and atrophy around the affected joint [16]. Muscles play a vital role in proprioception, as they sense changes in muscle length and tension. The weakness or dysfunction of muscles can negatively impact proprioceptive function. (5) Chronic pain is a common symptom of OA [17]. Pain can interfere with the brain’s ability to interpret proprioceptive signals correctly, leading to diminished proprioceptive awareness.

Several studies have drawn attention to a notable connection between osteoarthritis (OA) and the loss of proprioception [16,18,19,20,21,22,23,24,25]. The reduction in joint space, a fundamental characteristic of OA, has been linked to a decline in joint position sense [18]. Barrett et al. documented a decreased joint position sense in individuals with knee OA compared to those without OA [19]. Additionally, various studies have reported a loss of joint position sense, as measured through different techniques, in individuals with OA of the knee [16,21,22]. It is noteworthy that this relationship is bidirectional: not only can the loss of proprioceptive mechanisms contribute to the development of OA, but OA itself can also lead to a reduction in proprioception [16,20,21]. Thus, maintaining proper proprioceptive function can be essential for joint stability, coordinated movement, and injury prevention. Impaired proprioception in OA patients can lead to reduced joint control, an increased risk of falls, and altered movement patterns, further contributing to joint degeneration.

Despite the last decade having shown a proliferation of studies on proprioception in OA, there is a lack of a systematic overview of these studies. The purpose of the article is to fill this gap by conducting a systematic review of the existing literature on proprioception and mechanoreceptors and their connection to OA. This review aims to answer specific questions, such as the following: (1) What are the functions of proprioception in joints with OA, and which structures within the OA joint are most affected? (2) What methods measure proprioceptive accuracy in OA? (3) Do patients with OA have reduced proprioceptive accuracy compared to healthy controls? (4) What causes reduced proprioceptive accuracy in OA? (5) What is the specific role and significance of mechanoreceptors in OA proprioceptive deficit?

## 2. Materials and Methods

### 2.1. Eligibility Criteria

The PICOS model (population, intervention, comparison, outcomes, and study design) was used to set up this review: (1) studies that evaluated proprioceptive function and mechanoreceptors in OA patients (population), submitted or not, (2) to a specific intervention (interventions), (3) with or without a comparison group (comparisons), (4) that described proprioceptive function and mechanoreceptors in OA patients (outcomes), in (5) preclinical and clinical studies (study design). Studies from September 2013 to September 2023 were included in this review if they met the PICOS criteria. Studies evaluating (1) proprioceptive function and mechanoreceptors in non-OA patients, (2) mathematical modeling tool construction; (3) proprioceptive function and mechanoreceptors in physiological conditions; (4) proprioceptive function and mechanoreceptors in other pathological conditions different from OA; and (5) articles with partial data were excluded. Moreover, reviews, letters, comments to Editor, meta-analyses, case-reports, protocols and recommendations, editorials, guidelines, and articles not written in English were excluded. This review is not registered.

### 2.2. Search Strategies

The literature review involved a systematic search conducted in September 2023 according to the Preferred Reporting Items for Systematic Reviews and Meta-Analyses (PRISMA) statement [26]. The search was conducted on three databases: PubMed, Scopus, and Web of Science. The resulting combination of terms was used (proprioception OR proprioceptional OR proprioceptions OR proprioceptive OR proprioceptively) AND (mechanoreceptors OR mechanoreceptor) AND (osteoarthritis OR osteoarthritides) and for each of these terms, free words and managed vocabulary specific to each bibliographic database were merged using the operator “OR”. The combination of free vocabulary and/or Medical Subject Headings (MeSH) terms for the recognition of studies are also reported in Table 1.

### 2.3. Selection Process

Following the removal of duplicate articles using a public reference manager (Mendeley Desktop v.1.19.8), the pool of potentially relevant articles underwent initial screening based on their titles and abstracts, a process conducted by a single reviewer (FS). Any studies that did not meet the predefined inclusion criteria were excluded, and any uncertainties were addressed by involving a second reviewer (GG). Ultimately, the remaining studies were included in the final stage of data extraction.

### 2.4. Data Collection Process and Synthesis Methods

The process of data extraction and synthesis commenced with a systematic cataloging of the details contained within the studies under review. To enhance the validity of the process and to ensure that no potentially relevant findings were inadvertently overlooked during synthesis, a single author (F.S.) undertook the extraction task. This involved the creation of a comprehensive table, wherein various key elements were meticulously recorded. These elements included the type of study, the experimental design employed, the specific site of OA under investigation, the aspects of proprioceptive function and mechanoreceptors that were analyzed, the primary outcomes and findings, as well as the corresponding references for each study.

### 2.5. Risk of Bias Assessment

One reviewer (F.S.) analyzed the methodological quality of the included studies. The methodological quality of the clinical studies included in the analysis was assessed using the ROBINS-I tool, which was specifically designed to evaluate the risk of bias in non-randomized studies of interventions [27].

## 3. Results

### 3.1. Study Selection

**Figure 2:**
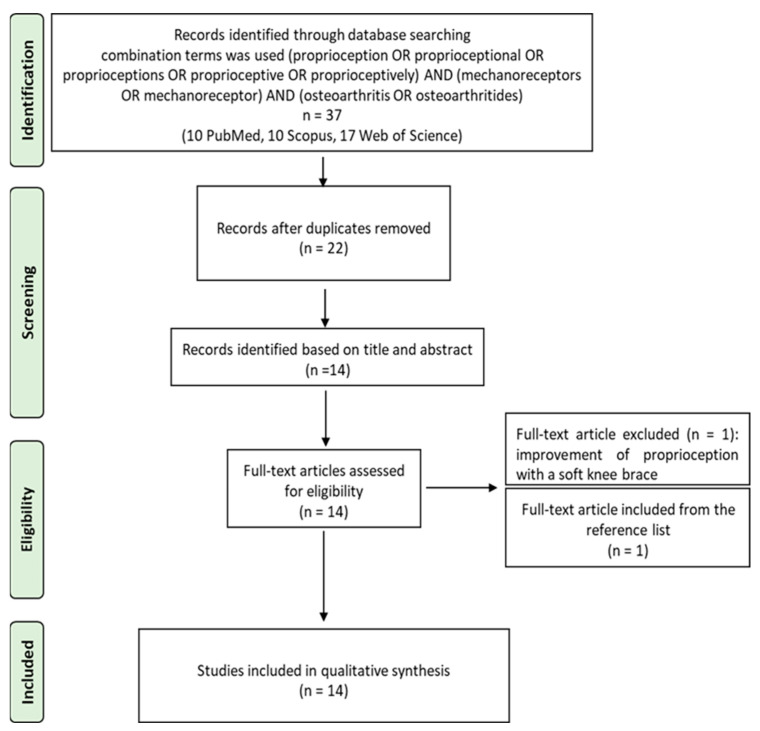
The PRISMA flow diagram for the systematic review detailing the database searches, the number of abstracts screened, and the full texts retrieved.

The initial search found 37 studies. Of those, 10 were detected using PubMed, 10 using Scopus, and 17 were found in Web of Science. Articles were uploaded in Mendeley Desktop version 1.17.9 to remove duplicates and the resulting 22 articles were screened for title and abstract. In total, 14 complete articles were reviewed to determine whether the publication met the inclusion criteria, and 13 were considered eligible for the review. From the reference lists of the selected articles one extra publication was found. Of the fourteen articles eligible for the review, seven were prospective, four were cross-sectional, one was retrospective, one case–control and one prognostic studies. Search strategy as well as study inclusion and exclusion criteria are specified in Figure 2.

### 3.2. Study General Characteristics

Table 2 describes the demographic characteristics of the studies included in the analysis. Out of 14 studies, 6 had a control group (non-OA), while the rest focused solely on cohorts of OA patients. The largest cohort consisted of 105 patients [28]. In total, 11 out of 14 studies had patient cohorts with 50 subjects or fewer, with the smallest cohort including 11 patients [29,30], while 3 studies had larger patient cohorts. OA was diagnosed prevalently using radiography, and the severity was defined using various grading scales, including Kellgren and Lawrence grades, Outerbridge grade, WOMAC score, Eaton stage, and Ahlbäck scale. The most common age group was 60–75 years. One study recruited younger participants in both the OA and healthy control groups [31]. Almost all the studies considered both female and male patients, with a higher prevalence of females. Two studies did not specify the sex [32,33]. Except for two studies [29,30] that evaluated the proprioceptive feedback mechanism in the OA carpometacarpal joint, all the other studies analyzed proprioception in the OA knee joint, particularly focusing on the posterior cruciate ligaments (PCLs) where the changes and presence of specific types of mechanoreceptors (Ruffini, Pacini, and Golgi-Mazzoni corpuscles) were evaluated.

### 3.3. Knee Proprioceptive Deficit

Çabuk et al. conducted a study to identify and quantify mechanoreceptors in the posterior cruciate ligament (PCL), anterior capsule (AC), and medial meniscocapsular junction (MCJ) in patients with knee OA [32]. They found that the numbers of Golgi corpuscles, Ruffini corpuscles, free nerve endings, total nerve endings, and small vessels in the PCL were low in the OA group. Similarly, the numbers of Golgi corpuscles, free nerve endings, and total nerve endings in the AC were reduced in the OA group. Comparable results were also obtained examining a larger cohort of OA patients [34]. Moreover, it was demonstrated that the number of PCL mechanoreceptors decreased with increasing WOMAC score (a measure of knee OA severity) [33]. The presence of neural structures in the PCL resected during posterior stabilized arthroplasty and during primary total knee arthroplasty were found, respectively, in ~67.5% and 77% of OA patients, with the neurovascular bundle being degenerated in 65% of the cases [35,36]. Nervous structures were found to be more frequently detected in knees with varus alignment compared to knees with valgus alignment, with a prevalence of 77% in varus knees as opposed to 50% in valgus knees, and severe histologic degeneration of the PCL correlated with neurovascular bundle degeneration. Oliveira et al. also quantified and compared these neural elements in the PCL of healthy and OA knees [37]. The overall mean area of the neural elements was 0.96 ± 0.67%, with the value in the healthy group being 1.02 ± 0.67% and 0.80 ± 0.64% in the OA group. Several findings were noted in the study. First, there was no observed correlation between the quantification of neural elements and the age of the individuals in the study [33,37]. However, within the OA group, a statistically significant reduction in neural elements was observed in males compared to females. Furthermore, there was no discernible difference in the quantification of neural elements between knees with varus alignment and those with valgus alignment within the OA group [37]. In addition to PCL, the anterior cruciate ligament (ACL) in knee OA also exhibited a reduction in the number of mechanoreceptors [38]. In detail, various types of nerve endings and different proportions of proprioceptors were identified in ACL, including Ruffini corpuscles (the main type), Pacini corpuscles (rarely found), Golgi-Mazzoni corpuscle (rarely found), and free nerve endings (second most common) [38].

Apart from histological and immunohistochemical analyses, other methods for measuring knee proprioception have been used. Using dynamic, single-leg, postural stabilometry, a significant proprioceptive deficit in patients with chondral injuries was detected compared to healthy controls [31]. A further proprioception deficit in knee of OA patients with a co-existing medial meniscal tear was detected by determining the joint motion detection threshold in the knee extension direction [28]. The motion sense of ankle/subtalar joints was also negatively affected in OA patients, although hip abduction and knee flexion motion sense were similar to subjects without knee OA [39]. It was observed that the proprioceptive impairments, as indicated by joint position testing, which are associated with knee OA, may be specific to the knee joint and not extend to other body regions affected by OA, such as the elbow and ankle [40]. Finally, a retrospective study on 45 OA patients compared two arthroplasty designs (cruciate substituting vs. cruciate retaining) in terms of proprioception (postural control and balance) and found that proprioceptive capacities in total knee arthroplasty (TKA), in fact, recover to at least the state of the non-operated side, but the PCL does not seem to contribute significantly to this recovery [41].

### 3.4. Proprioceptive Deficit in Joints Other Than Knee

Out of the fourteen studies that were reviewed, only two examined joints other than the knee. These studies focused on the anterior oblique (AOL) and dorsal radial ligament (DRL) of the first carpometacarpal joint (CMC1) in individuals with and without osteoarthritis (OA) [29,30]. Interestingly, they discovered the presence of mechanoreceptors in the CMC-1 ligaments of all patients with OA, with the DRL showing notably higher innervation compared to the AOL. Furthermore, in the CMC-1 ligaments of patients who underwent surgery for CMC-OA, various mechanoreceptor types were identified, including Ruffini Endings, Pacini corpuscles, and corpuscles that could not be classified.

### 3.5. Risk of Bias Assessment

**Figure 3:**
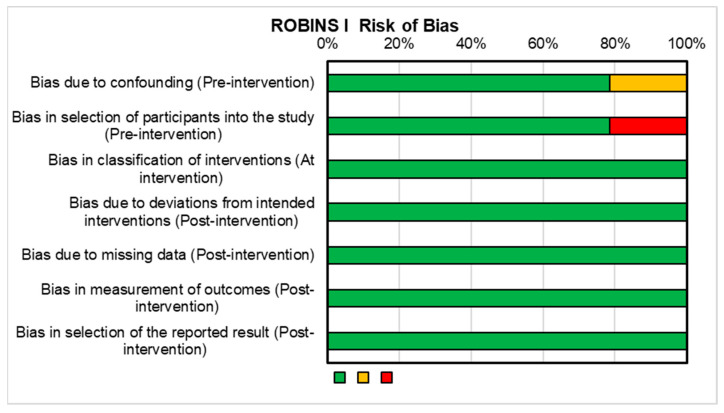
ROBINS-I tool for assessing risk of bias in non-randomized clinical studies. Green: no risk of bias; Yellow: moderate risk of bias; Red: High risk of bias.

A risks of bias assessment is reported in Figure 3. For these studies, the risk of bias was mainly low, with only three study in which two domains presented a risk. The two domains were ‘bias due to confounding’, which present a moderate risk, and ‘selection of participants into the study in pre-intervention’, which has a high risk.

**Table 2:** Main characteristics of clinical studies included in the review.

Study Type	Sample Size Mean Age	Gender Male/Female	OA Site	OA Severity	Analyzed Location	Proprioceptive Function and/or Mechanoreceptors Analyzed	Findings	Ref.
Prospective	Chondral injuries pts n = 8 34 ± 9 Control pts n = 50 25 ± 5	Chondral injuries pts 6:2 Control pts 35:15	Knee	Outerbridge grade: 3–4	Cartilage	Dynamic postural stabilometry, Patient-reported outcome measures (PROMs)	Significant proprioceptive deficit in chondral injuries group versus control group as well as in all PROMs	Al-Dadah et al., 2020 [31]
Prognostic	OA pts n = 30 66.4 ± 7.5 Control pts n = 10 67.6 ± 6.5	Not reported	Knee	KL score: 3–4	PCL AC MCJ	PCL degeneration evaluated with HE; types and numbers of mechanoreceptors evaluated with S100 immunostaining	Low numbers of mechanoreceptors in patients with OA in the PCLs and ACs	Çabuk et al., 2017 [32]
Cross-sectional	OA pts Group A n = 8 60–69 yrs Group B n = 12 70–79 yrs Group C n = 8 ≥80 yrs	Not reported	Knee	WOMAC score: group I ≤80; n = 8 group II 81–120 n = 10 group III >120 n = 10	PCL	HE and S-100 immunohistochemical staining	Number of mechanoreceptors in the PCL decreased significantly with higher (worse) WOMAC score	Chen et al., 2023 [33]
Prospective	OA pts n = 57 66.48	20:37	Knee	Not reported	ACL	HE, Van Gison, and S100 immunohistochemical staining	Low number of proprioceptors in the OA ACL	Gerasimov et al., 2019 [38]
Prospective	OA pts n = 11 67.0	1:10	CMC1	Eaton stage	AOL and DRL	DAPI, p75, and PGP9.5 immunohistochemical staining	Identification of a unique type and distribution of mechanoreceptors in the OA CMC1	Ludwig et al., 2015 [29]
Cross-sectional	OA pts n = 26 63.0 ± 7.5 Control pts n = 26 63.0 ± 10.9	OA pts 8:18 Control pts 8:18	Knee	KL score: 2	Knee	Proprioceptive sensory measurements through isokinetic dynamometer	Hip abduction and knee flexion motion sense like the without knee OA subjects; motion sense of ankle/subtalar joints were negatively affected in OA patients	Mani et al., 2020 [39]
Prospective	OA pts n = 50 70.7 (53–84) Control pts n = 10	OA pts 10:40 Control pts 3:7	Knee	Ahlbäck scale: n = 20 grade 3 n = 19 grade 4 n = 11 grade 5	PCL	HE, Alcian blue, Gomori, van Gieson, and S100 immunohistochemical staining	A close correlation between the severity of degenerative changes on the X-ray images according to the Ahlbäck scale, and the presence of proprioceptors of PCLs	Marczak et al., 2016 [34]
Cross-sectional	OA pts n = 34 Range: 53 to 87 yrs	9:22	Knee	Ahlbäck scale: n = 26 grade 1–3 n = 8 grade 4–5	PCL	HE and Gomori, and S100 immunohistochemical staining	Severe PCL degeneration related to neurovascular bundle compromise. Intrinsic neural structures detected in most of the PCL of patients submitted to knee arthroplasty for OA	Martins et al., 2015 [35]
Prospective	OA pts n = 11 67.0	1:10	CMC-1	Eaton stage: 2–4	AOL and DRL	HE and DAPI, and p75 and PGP9.5 immunohistochemical staining	The dense collagen structure and rich innervation of the DRL in patients with OA suggest that the DRL has an important proprioceptive and stabilizing role	Mobargha et al., 2014 [30]
Case–control	OA pts n = 14 71.1 ± 8.4 Control pts n = 24 59.8 ± 24.4	OA pts 3:11 Control pts 13:11	Knee	Not reported	PCL	HE and S100 immunohistochemistry	Decrease in neural element quantification in PCL of OA patients in relation to non-OA	Oliveira et al., 2021 [37]
Prospective	OA pts n = 62 67.0 ± 11.0	16:46	Knee	Not reported	PCL	HE, Alcian blue, and Masson trichrome and S100 immunohistochemistry	Retaining the PCL in total knee replacement is a good option for better kinematics, stability, and proprioception	Rajgopal et al., 2014 [36]
Prospective	OA pts n = 30 66 ± 7 Control pts n = 30 65 ± 8	OA pts 13:17 Control pts 13:17	Knee, ankle, and elbow	KL grade: 3–4	Knee, ankle and elbow	Joint position testing	Proprioceptive impairments associated with knee OA may be localized to the knee joint and not generalized to other body regions	Shanahan et al., 2015 [40]
Cross-sectional	OA pts n = 105 61.4 ± 6.9	32:73	Knee	KL score: n = 1 grade 0 n = 31 grade 1 n = 28 grade 2 n = 26 grade 3 n = 19 grade 4	Entire joint	Joint motion detection threshold in the knee extension direction	Reduced proprioceptive accuracy associated with both the number of regions with meniscal abnormalities and the extent of abnormality	Van der Esch et al., 2013 [28]
Retrospective	OA patients n = 45 (n = 18 with cruciate substituting—PS-, and n = 27 with cruciate-retaining—CR- design) CR: 70.5 ± 6.4 PS: 68.0 ± 8.4	male/female ratio: CR: 0.33 PS: 0.55	Knee	Not reported	PCL	Balance and postural control using the Balance Master system	Retaining the PCL does not result in an improved proprioception	Vandekerckhove et al., 2015 [41]

Patients: pts; KL: Kellgren and Lawrence grades; PCL: posterior cruciate ligament; AC: anterior capsule; MCJ: medial meniscocapsular junction; HE: hematoxylin–eosin; ACL: anterior cruciate ligament; CMC1: first carpometacarpal joint; AOL: anterior oblique ligament; DRL: dorsal radial ligament; DAPI: 4′,6′-diamidino-2-phenylindole; years: yrs.

## 4. Discussion

In this review, we found that, except for two studies that evaluated the proprioceptive feedback mechanism in the OA carpometacarpal joint, all the other studies assessed proprioception in the OA knee joint, with a particular focus on the PCL (posterior cruciate ligament). The knee is among the most affected joints, and as OA worsens, the PCL gradually degenerates due to the invasion of inflammatory factors and physical wear over time [42,43,44,45]. The PCL is a critical ligament within the knee joint, primarily responsible for preventing the posterior translation of the tibia relative to the femur. Additionally, it contributes significantly to the knee joint’s dynamic stability through proprioception and muscle engagement [46]. In their study, Çabuk et al. [32] observed that the nerve tissue in the posterior cruciate ligament (PCL) of patients with OA was less abundant compared to healthy patients. However, the authors did not analyze the impact of the varying degrees of OA on the number of mechanoreceptors. Kleinbart et al. [47] confirmed that OA can exacerbate histological degeneration in the PCL. Furthermore, Levy et al. [48] reported a steady increase in the histological degeneration of the PCL with worsening Outerbridge cartilage injury classifications (grades 0–4), progressing from grade 0 to 3. Cartilage injury can be considered a manifestation of OA; hence, it is logical that the histological degeneration of the PCL worsens with OA progression. Martins et al. [35] also noted that as histological degeneration advanced, the neurovascular structure of the PCL suffered greater damage. These data underline that the proprioceptive accuracy of the PCL is impaired in knee OA patients. Several factors related to knee OA have been hypothesized as possible causal factors for impaired proprioceptive accuracy, particularly mechanoreceptors alterations. Changes in the presence of specific types of mechanoreceptors (Ruffini, Pacini, and Golgi-Mazzoni corpuscles) were evaluated in most studies (9/14) using a combination of immunohistochemistry and histology (S-100, p75, PGP9.5, and hematoxylin–eosin staining) [28,29,30,31,32,33,34,35,36,37,38,39,40,41]. It was found that the numbers of Golgi corpuscles, Ruffini corpuscles, free nerve endings, total nerve endings, and small vessels in the PCL were low in OA patients [28,29,30,31,32,33,34,35,36,37,38,39,40,41]. Thus, dysfunctional articular mechanoreceptors, prevalent in severe OA knees, may lead to impaired proprioceptive accuracy. Furthermore, some studies also detected that the number of PCL mechanoreceptors decreased with an increasing WOMAC score (a measure of knee OA severity) [31]. Immunohistochemistry and histology are a valuable tool for the analysis of Ruffini, Pacini, and Golgi-Mazzoni corpuscles, as they provide detailed information about protein expression and morphology. However, researchers should be aware of the limitations and challenges associated with these techniques, especially in terms of specificity, sample preparation, and quantitative analysis. In this context, could be beneficial to complement immunohistochemistry and histology with other techniques to obtain a more comprehensive understanding of these sensory receptors. In fact, in addition to histological and immunohistochemical analyses, other methods, such as the stabilometric study of the static dynamic position, for measuring knee proprioception were also used in the studies analyzed in this review [28,31,39,40,41]. Using dynamic stabilometry, a significant proprioceptive deficit was also detected in patients with isolated articular cartilage lesions of the knee, indicating that articular cartilage lesions also have a considerable impact on knee proprioceptive function [31]. Thus, the results of these studies further indicated that a defined proprioceptive deficiency exists in patients with knee OA. However, it is important to highlight that stabilometry and isokinetic dynamometry do not provide direct measures of proprioception, but they can reveal balance impairments and difficulties in maintaining stability, which may be indicative of underlying proprioceptive deficits.

As shown in our review for OA disease, the presence of proprioceptive mechanoreceptors in the context of capsule–ligamentous joint structures has been amply demonstrated in recent decades, but their presence and characterization within the cartilaginous layer has not yet been clearly highlighted to the best of our current knowledge [49,50,51,52]. Improving our understanding of articular cartilage functions, as well as characterizing proprioceptive mechanoreceptors also within the cartilaginous or subchondral layers could be of crucial importance for developing specific treatments for proprioceptive deficits. To date, considering the role of proprioception in OA, various modalities and treatments can be employed to improve proprioceptive deficits, including proprioceptive exercises, like balance training and joint position sense training; strengthening exercises; neuromuscular electrical stimulation; manual therapy techniques; bracing or orthotics; sensory training with methods like vibration and biofeedback; mind–body practices, like Tai Chi and yoga; aquatic therapy; cognitive training; and patient education about joint protection and lifestyle modifications [53,54,55,56]. Medications and supplements, like nonsteroidal anti-inflammatory drugs and glucosamine/chondroitin, may also help by reducing pain and inflammation, potentially enhancing proprioceptive feedback [53,54,55,56]. Consulting with a healthcare provider or physical therapist is essential to develop a personalized proprioceptive training program and select the most appropriate modalities and treatments for addressing proprioceptive deficits in OA.

A limitation of this systematic review is its descriptive approach. No meta-analysis of the included articles was performed since the presence of statistically significant heterogeneity between them; therefore, definitive conclusions cannot be drawn. Nevertheless, we assume that this narrative review provides a comprehensive overview of the current state of knowledge regarding the role of proprioceptive accuracy in OA. Furthermore, it highlights areas in need of future research, such as the following: (1) proprioceptive feedback mechanisms in OA joints other than the knee, in regard to which there are some ongoing studies with the aim of evaluating the proprioceptive response in patients with ankle OA; (2) proprioceptive function of the contra-lateral uninjured joint; (3) whether a proprioceptive deficit causes OA or occurs as a consequence of it; (4) the relationship between age at OA onset and mechanoreceptor deficit; (5) the quantitative relationship between proprioception and mechanoreceptors; and (6) the relationship between proprioception, OA disease, and symptoms [39,52].

In conclusion, considering the increased susceptibility of aging populations to developing OA and the immense global burden of the disease, the role of proprioception in OA should be further explored. The available data strongly indicate that collaborative efforts among researchers in the field to gain a deeper understanding of fundamental proprioceptive mechanisms, and to investigate the hypothesis that impaired proprioception, in a broader context, might increase the susceptibility to joint injuries and contribute to the progression of joint damage, could have a profoundly beneficial impact on mitigating the burden of this disease. Understanding which neural substrates and other factors need to be specifically targeted to achieve optimal clinical and surgical outcomes in the event proprioception is impaired in OA patients also needs to be uncovered.

## Figures and Tables

**Table 1:** Combination of free-vocabulary and/or Medical Subject Headings (MeSH) terms for the identification of studies in PubMed, Scopus, and Web of Science.

PubMed	(“proprioception“[MeSH Terms] OR “proprioception”[All Fields] OR “proprioceptional”[All Fields] OR “proprioceptions”[All Fields] OR “proprioceptive”[All Fields] OR “proprioceptively”[All Fields] OR “proprioceptivity”[All Fields]) AND (“mechanoreceptors”[MeSH Terms] OR “mechanoreceptors”[All Fields] OR “mechanoreceptor”[All Fields]) AND (“osteoarthritis”[MeSH Terms] OR “osteoarthritis”[All Fields] OR “osteoarthritides”[All Fields]) AND (2013:2023[pdat])
Scopus	(TITLE-ABS-KEY (proprioception) OR TITLE-ABS-KEY (proprioception) OR TITLE-ABS-KEY (proprioceptions) OR TITLE-ABS-KEY (proprioceptive) OR TITLE-ABS-KEY (proprioceptive) OR TITLE-ABS-KEY (proprioceptive) AND TITLE-ABS-KEY (mechanoreceptors) OR TITLE-ABS-KEY (mechanoreceptor) AND TITLE-ABS-KEY (osteoarthritis) OR TITLE-ABS-KEY (osteoarthritis)) AND PUBYEAR > 2012 AND (LIMIT-TO (LANGUAGE, “English”)) AND (LIMIT-TO (DOCTYPE, “ar”))
Web of Science	(TS = proprioception OR TS = proprioceptional OR TS = proprioceptions OR TS = proprioceptive OR TS = proprioceptively) AND (TS = mechanoreceptors OR TS = mechanoreceptor) AND (TS = osteoarthritis OR TS = osteoarthritides)—with Publication Year from 2013 to 2023, English

## Data Availability

Not applicable.
